# Pressure transmitting device: a simple and safe method of continuous aspiration of subglottic secretions during orotracheal intubation

**DOI:** 10.1186/cc10190

**Published:** 2011-06-22

**Authors:** CES Almeida, C Saghabi, AR Marra

**Affiliations:** 1 Hospital Israelita Albert Einstein, São Paulo - SP, Brazil

## Introduction

**Figure 1:**
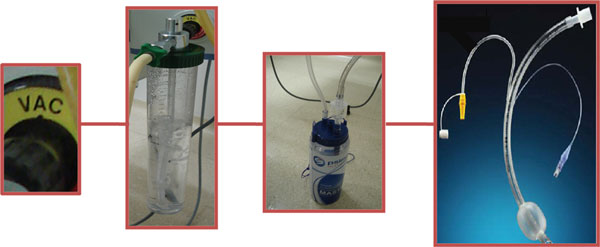


Many interventions are known to decrease the incidence of ventilator-associated pneumonia, which has great impact on mortality, length of stay and costs in intensive care units. One of them is the aspiration of the secretions that pool above the cuff of the endotracheal tube [1]. It is a simple device but its use is not free from complications [2], being, most of them, bleedings and obstructions due to lesions of tracheal mucosa. The maintenance of a constant suction, without wide pressure variation, is an important point to minimize these complications. The common manometers do not have enough precision to set an adequate aspiration pressure, because of its broad scale, and are not able to avoid or to limit pressure variations in case of partial occlusions, by secretion, for example, facilitating lesions occurrence. Pressure transmitting devices (Figure 1), usually used for continuous aspiration of pleural drainage, have those helpful characteristics. It can be set in an adequate aspiration pressure (20 mmHg ~ 27 cmH_2_O) by setting the water column height. It avoids suction pressure variations since the air bubbles up on the water, balancing pressure inside the system.

## Methods

Pressure transmitting devices were tested in 12 patients with subglottic aspiration on their orotracheal tubes. They were watched for complications and the findings are reported. The aspiration pressure used was set at 20 cmH_2_O.

## Results

The proposed system was used for periods that lasted from 3 to 14 days in each patient. It was able to remove the subglottic secretions in all tested cases. There were two episodes of system obstruction due to thick secretions, one of them was a blood clot (the patient had an abundant oral bleeding), easily treated with gentle suction using a 5-ml syringe. There was one case of obstruction resolved with air injection through the subglottic suction lumen. There was no bleeding related to subglottic suction. There was no ventilator-associated pneumonia.

## Conclusion

In those reported cases, the subglottic suction system using a pressure transmitting device seemed to be effective, without serious complications. This study of cases is not able to affirm these conclusions. It is just an initial test of a new method. For better evidence, this system has to be compared with other devices, like manometers, that are usually used for aspiration pressure control.
